# The Molecular Subtype Classification Is a Determinant of Sentinel Node Positivity in Early Breast Carcinoma

**DOI:** 10.1371/journal.pone.0020297

**Published:** 2011-05-31

**Authors:** Fabien Reyal, Roman Rouzier, Berenice Depont-Hazelzet, Marc A. Bollet, Jean-Yves Pierga, Severine Alran, Remy J. Salmon, Virginie Fourchotte, Anne Vincent-Salomon, Xavier Sastre-Garau, Martine Antoine, Serge Uzan, Brigitte Sigal-Zafrani, Yann De Rycke

**Affiliations:** 1 Department of Surgery, Institut Curie, Paris, France; 2 Department of Gynaecology, Hôpital Tenon, Paris, France; 3 Assistance Publique-Hôpitaux de Paris, Université Pierre et Marie Curie, Paris 6, Paris, France; 4 Department of Medical Oncology, Institut Curie, Paris, France; 5 Department of Radiation Oncology, Institut Curie, Paris, France; 6 Department of Tumour Biology, Institut Curie, Paris, France; 7 Department of Pathology, Hôpital Tenon, Paris, France; 8 Department of Biostatistic, Institut Curie, Paris, France; National Cancer Institute, United States of America

## Abstract

**Introduction:**

Several authors have underscored a strong relation between the molecular subtypes and the axillary status of breast cancer patients. The aim of our work was to decipher the interaction between this classification and the probability of a positive sentinel node biopsy.

**Materials and Methods:**

Our dataset consisted of a total number of 2654 early-stage breast cancer patients. Patients treated at first by conservative breast surgery plus sentinel node biopsies were selected. A multivariate logistic regression model was trained and validated. Interaction covariate between ER and *HER2* markers was a forced input of this model. The performance of the multivariate model in the training and the two validation sets was analyzed in terms of discrimination and calibration. Probability of axillary metastasis was detailed for each molecular subtype.

**Results:**

The interaction covariate between ER and *HER2* status was a stronger predictor (p = 0.0031) of positive sentinel node biopsy than the ER status by itself (p = 0.016). A multivariate model to determine the probability of sentinel node positivity was defined with the following variables; tumour size, lympho-vascular invasion, molecular subtypes and age at diagnosis. This model showed similar results in terms of discrimination (AUC = 0.72/0.73/0.72) and calibration (HL p = 0.28/0.05/0.11) in the training and validation sets. The interaction between molecular subtypes, tumour size and sentinel nodes status was approximated.

**Discussion:**

We showed that biologically-driven analyses are able to build new models with higher performance in terms of breast cancer axillary status prediction. The molecular subtype classification strongly interacts with the axillary and distant metastasis process.

## Introduction

Gene expression profiling of invasive breast carcinoma has resulted in highlighting three main categories of breast cancer with very specific features [luminal-like, basal-like, HER2-like][1]. Wirapati et al [2] showed that three main vectors-genes [*ESR1*, *HER2* and *STK6, a marker of proliferation*] are the biological backbone of this classification. Although the methodology to determine the molecular subtypes has still to be improved[3], many publications have validated this classification [2] [4]. It has been shown that the molecular subtypes differ in their response to neaoadjuvant systemic treatment [5], loco-regional recurrence [6], metastasis pattern [7], [8], time to metastasis and overall survival [3]. Furthermore, several authors have underscored a strong relation between the molecular subtypes classification and the axillary status of breast cancer patients [9]–[16]. As the nodal status is the most robust and the strongest factor correlated to overall survival in breast cancer patients, and is one of the major determinants in therapeutic decisions, axillary staging (either by sentinel node biopsy or axillary lymph node dissection) is a mandatory step in breast cancer management. Many predictors of axillary lymph nodes metastases have been previously published. Tumour size, tumour grade, tumour location, presence of lymphatic/vascular invasion, high MIB-1 index, age at diagnosis, S phase, estrogen receptor status (ER), progesteron receptor status (PR), *HER2* status are independent variables identified in these studies [17]–[25].

The aim of our work was to decipher the relation between the molecular subtype classification as defined by a combination of *ER* and *HER2* status evaluated by immuno-histochemistry (IHC) and confirmed by FISH in case of IHC-HER2 2+ and the probability of a positive sentinel node biopsy. Using one training set and two validation sets we showed a benefit to introduce the *ER* and *HER2* biomarkers interaction covariate to identify, before surgery, a patient with a high risk of axillary metastasis. Furthermore we showed for each molecular subtype a very specific correlation pattern between the tumour size and the probability of a positive sentinel node biopsy. We hypothesized from these results that the axillary lymph node metastasis process is predominantly correlated to intrinsic biological properties in the ER negative *HER2* negative breast cancer subgroup whereas stochastic events, tumour size, growth rate and lympho-vascular invasion are the main determinants in the ER positive or *HER2* positive breast cancer subgroups.

## Materials and Methods

### Patients

Our dataset consisted of one training set of 1543 early-stage breast cancer patients treated between 2000 and 2007 and a validation set of 615 early-stage breast cancer patients treated in 2008 and identified through the Institut Curie prospective breast cancer database. A second external validation set consisted of 496 early-stage breast cancer patients treated between 2001 and 2007 and identified through the Hopital Tenon, department of gynecology, prospective breast cancer database. The main inclusion criterion were patients with an infiltrating breast carcinoma <30 mm based on clinical and radiological features, normal physical examination of the axilla, treated at first by conservative surgery plus a sentinel node (SN) biopsy. The procedure was performed with blue patent, radioisotope or a combination, as previously described, in line with French recommendations. SN biopsy was performed as previously described [26]. Axillary lymph node dissection was performed during the same procedure when the SN was positive by imprint cytology or frozen section. A second operation was performed when either hematoxylin-eosin staining or immunohistochemistry revealed tumor cells in the SN postoperatively, including isolated tumour cells. Pathologic SN examination methods were as reported previously [26]. Patients receiving a neoadjuvant treatment (chemotherapy, hormone-therapy or radiotherapy) or with a locoregional recurrence were systematically excluded from the study. The clinical data (age at diagnosis, treatment protocols) were extracted from the Institut Curie prospective breast cancer database and from the Hospital Tenon, department of gynecology, prospective breast cancer database.

### Tumor samples

The following histological features were retrieved: tumour type, tumour size, histological grade according to Elston and Ellis grading system (Histopathology 1991), Mitotic Index, Lympho Vascular Invasion, Estrogen Receptor status, Progesterone Receptor status, *HER2* status, number of positive sentinel nodes, number of sentinel nodes. Mitotic Index (MI) corresponded to the number of mitoses observed in 10 successive high power fields (HPF) using a microscope with a 40x /0.7 objectives and a 10x ocular, each. Mitotic Index was assessed on histological sections stained by Hematein, Eosin and Saffron. The criteria of Van Diest and al were used to define mitotic figures [27], [28]. Estrogen Receptor (ER) and Progesteron Receptor (PR) immunostainings were determined as follow. After rehydration and antigenic retrieval in citrate buffer (10 mM, pH 6.1), the tissue sections were stained for estrogen receptor (clone 6F11, Novocastra, 1/200), and progesterone receptor (clone 1A6, Novocastra, 1/200). Revelation of staining was performed using the Vectastain Elite ABC peroxidase mouse IgG kit (Vector Burlingame, CA) and diaminobenzidine (Dako A/S, Glostrup, Denmark) as chromogen. Positive and negative controls were included in each slide run. Cases were considered positive for ER and PR according to standardized guidelines using ≥10% of positive nuclei per carcinomatous duct. The determination of *HER2* over-expression status was determined according to the American Society of Clinical Oncology (ASCO) guidelines [29].

The SLN histopathological assessment protocol has been published by Fréneaux et al [26]. SLN samples were serially sectioned and stained with HE. Negative HE cases were then analyzed by serial sectioning with IHC. Positive sentinel nodes were classified into two groups according to the size of the metastasis: macrometastasis (>2 mm) and micrometastasis (< = 2 mm) detected either by HE staining or by cytokeratin IHC.

### Statistical model

Baseline characteristics were compared between groups using Chi-square or Fisher's exact tests for categorical variables and Student's t-tests for continuous variables. To develop well-calibrated and exportable nomograms for prediction of sentinel node positivity, we built a multivariate logistic regression model in a training cohort and validated it in two independent validation cohorts. First, univariate logistic regression analysis was performed to test the association of the sentinel lymph node status to the following variables: patient age, tumor diameter, histologic type of tumor, histological grade, lymphovascular invasion, ER status, PR status, HER2 status. Interaction covariate between ER and HER2 status were tested. The log-linearity of the continuous variables was study by fitting a polynomial functions with different degree or step functions in a logistic model. Age at diagnosis was subdivided in 3 classes and the tumour size was kept as a continuous variable. Second, a multivariate logistic regression analysis was performed to determine the probability of having a positive sentinel node biopsy procedure and to build a nomogram. Significant variables identified through univariate analysis were used as input in the multivariate analysis. The multivariate model performance was quantified with respect to discrimination and calibration. Discrimination (i.e., whether the relative ranking of individual predictions is in the correct order) was quantified with the area under the receiver operating characteristic curve. Calibration (i.e., agreement between observed outcome frequencies and predicted probabilities) was studied with graphical representations of the relationship between the observed outcome frequencies and the predicted probabilities (calibration curves): the grouped proportions versus mean predicted probability in groups defined by deciles and the logistic calibration were represented. The calibration was tested using the Hosmer-Lemeshow test. This test compares mean predicted probability and observed proportions using a 8 degree of freedom chi-square for the training set and a 9 degree of freedom chi-square for the validation sets. The analyses were performed using R software (http://cran.r-project.org).

A java web based interface is available at www.cancerdusein.curie.fr

The study was approved by the breast cancer study group of the Institut Curie.

## Results

**Figure 1:**
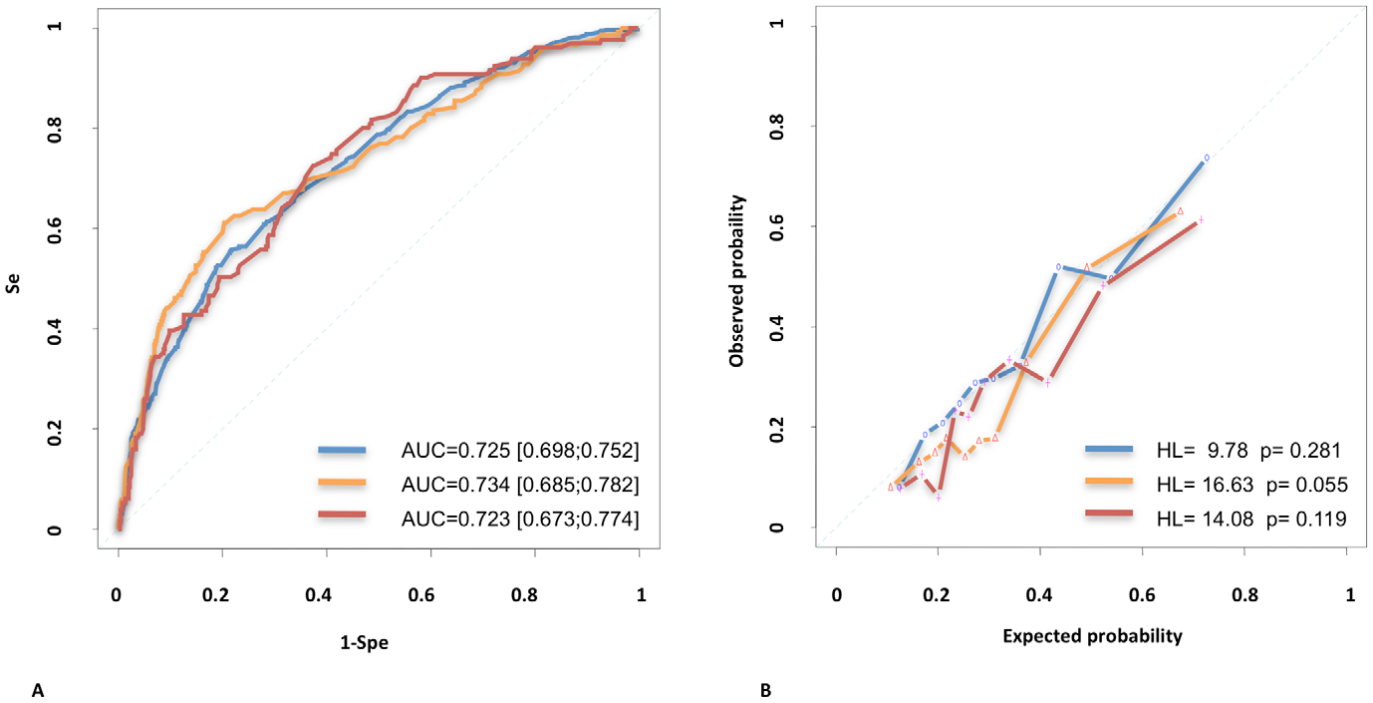
Receiver Operating Curves and Discrimination Curves. A) Receiver Operating Curves. Blue; Institut Curie Training Set. Orange; Institut Curie Validation Set. Red; Hopital Tenon Validation Set. Se; Sensitivoty. Spe; Specificity. B) Discrimination Curves. Blue; Institut Curie Training Set. Orange; Institut Curie Validation Set. Red; Hopital Tenon Validation Set.

**Figure 2:**
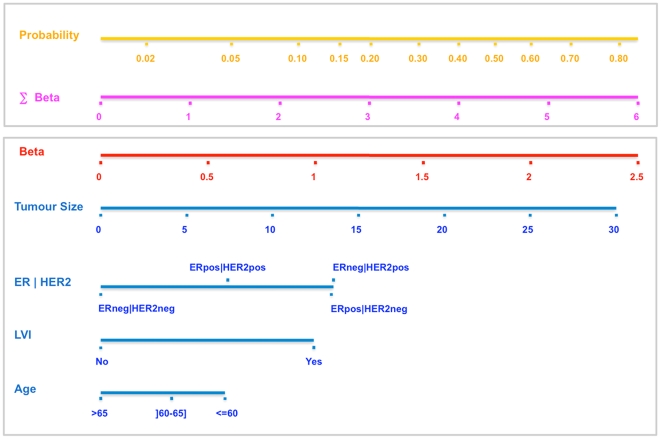
Nomogram to calculate the probability of sentinel node positivity in breast carcinoma. Nomogram to calculate the probability of sentinel node positivity in breast carcinoma. First: identify for each of the 4 variables the corresponding Beta value. Second, calculate the sum of the 4 Beta values. Third, report the Sum Beta Value to the Probability scale.

**Figure 3:**
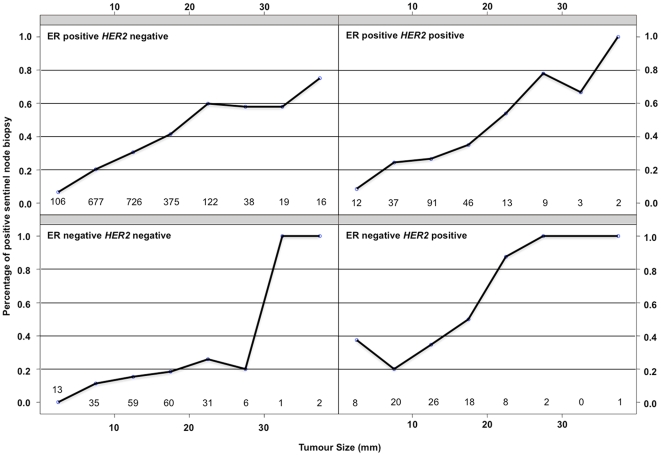
Percentage of positive sentinel node. Percentage of positive sentinel node calculated for each 5 mm tumour size subclasses from 0 to 40 mm. Number of patient by tumour size subclasses are printed. The training and two validation datasets have been merged to determine these probability plots.

Table 1 summarizes the training (1543 patients) and the two validation sets (615 and 496 patients). These three populations significantly differ in terms of age at diagnosis, ER status, HER2 status, histological grade, lympho vascular invasion, histological subtypes, number of sentinel nodes removed and number of positive sentinel node biopsy. These differences are of major interest in a validation process to test the robustness of a classification algorithm. The training set (Table 2) was composed of 516 patients with a positive sentinel node biopsy (33.4%) and 1027 patients with a negative sentinel node biopsy (66.6%). We showed that patients with a positive sentinel node biopsy differed from those with a negative biopsy in terms of age at diagnosis, ER status, pathological tumor size, histological grade, mitotic index, lympho vascular invasion and number of sentinel node removed. The proportion of patients with a positive HER2 status was not significantly different between the two groups [8.6% vs 7.6%, p = 0.58]. The interaction covariate between ER and HER2 status [ERneg HER2neg, ERpos HER2neg, ERpos HER2pos, ERneg HER2pos] was a stronger predictor (p = 0.0031) of positive sentinel node biopsy than the ER status by itself (p = 0.016). We designed a multivariate logistic regression model to determine the probability of having a positive sentinel node biopsy (Table 3). The initial input was based on the variables found significant in the univariate analysis. Tumour size, lympho-vascular invasion, molecular subtypes classification as defined by the interaction covariate between the ER and HER2 status and age at diagnosis were the final input into this model. Odds Ratio, Confidence Intervals and pvalue are summarized in table 3. The logistic regression parameters indicate the relative degree to which each of these variables is correlated to nodal metastasis. The performance of the multivariate model in the training and the two validation sets was analyzed in terms of discrimination and calibration. ROC curves are plotted in figure 1a. It showed a very similar area under curves (AUC) for each population [Training set AUC = 0.72 (95% CI, 0.69–0.75), IC validation set AUC = 0.73 (95% CI, 0.68–0.78), T validation set AUC = 0.72 (95% CI, 0.67–0.77)]. Calibration curves are plotted in figure 1b. The logistic model was well calibrated, with no significant difference between the predicted and the observed probability in the training and the two validation sets. The Hosmer-Lemeshow goodness of fit statistic showed similar results when applied to each datasets (Institut Curie Trainin Set p = 0.28, Institut Curie Validation Set p = 0.05, Hopital Tenon Validation Set p = 0.11). Using the multivariate logistic regression model, a nomogram was build (Figure 2). Finally we analyzed the correlation between the tumour size and the probability of having a positive sentinel node biopsy procedure for each molecular subtype (Figure 3, Table 4). We showed an almost null slope of the correlation axis in the ER negative HER2 negative subgroup. The probability of having an axillary metastasis seemed to be more or less 20% whatever the tumour size. Both ER positive (either HER2 negative or positive) tumour subgoups showed an intermediate slope and the ER negative HER2 positive tumour subgroup showed the steepest slope. Tumour size was a major determinant of axillary metastasis development only in the HER2 positive or ER positive tumour subgroups. Sentinel node biopsies for breast cancers of less than 30 mm was associated with a rate of less than 30% of axillary metastasis in the ER negative HER2 negative subgroup and with one higher than 50% in the other three subgroups. For each molecular subtype as defined by a combination of ER and *HER2* immuno-histochemistry markers, we summarized (table 5) eight publications addressing the percentage of axillary metastases [9]–[16].

**Table 1:** Clinical and pathological features of the training set and two validation sets.

	Training set *Number(%)*	Validation set 1 *Number(%)*	Validation set 2 *Number(%)*	Chi^2^ p value
**All patients**	1543 (100)	615 (100)	496 (100)	
**Age (years)**				
<60	973 (63.1)	330 (53.7)	296 (59.7)	1e-04
60–65	218 (14.1)	90 (14.6)	60 (12.1)	
>65	352 (22.8)	195 (31.7)	140 (28.2)	
**ER (IHC)**				
Positive	1343 (87)	557 (90.6)	458 (92.3)	0.0015
Negative	200 (13)	58 (9.4)	38 (7.7)	
***HER2*** ** (IHC-FISH)**				
Positive	132 (8.6)	47 (7.6)	125 (25.2)	<0.0001
Negative	1411 (91.4)	568 (92.4)	371 (74.8)	
**ER ** ***HER2*** **(IHC-FISH)**				
ERneg HER2neg	147 (9.5)	40 (6.5)	24 (4.8)	
ERpos HER2neg	1264 (81.9)	528 (85.9)	347 (70)	<0.001
ERpos HER2pos	79 (5.1)	29 (4.7)	109 (22)	
ERneg HER2pos	53 (3.4)	18 (2.9)	16 (3.2)	
**Tumour size (mm)**				
≤20	1333 (87.7)	560 (91.2)	440 (88.7)	NS
>20	187 (12.3)	54 (8.8)	56 (11.3)	
**Histological Grade**				
1	507 (33.1)	198 (32.4)	236 (47.6)	<0.0001
2	707 (46.1)	294 (48)	191 (38.5)	
3	318 (20.8)	120 (19.6)	67 (13.5)	
**Mitotic index**				
1	1048 (68.9)	437 (71.3)	344 (69.3)	NS
2	237 (15.6)	89 (14.5)	79 (15.7)	
3	235 (15.5)	87 (14.2)	62 (12.5)	
**LVI**				
Negative	1164 (76.4)	502 (81.6)	386 (77.8)	0.0301
Positive	360 (23.6)	113 (18.4)	110 (22.2)	
**Subtype**				
Ductal	1293 (83.8)	520 (84.6)	425 (85.7)	0.0037
Lobular	203 (13.2)	80 (13)	71 (14.3)	
Other(s)	47 (3)	15 (2.4)		
**No SN removed**				
1	416 (27)	126 (20.5)	153 (30.8)	1e-04
2–3	790 (51.2)	299 (48.6)	269 (54.2)	
>3	337 (21.8)	190 (30.9)	74 (15)	
**No of positive SN Biopsy**				
Positive	516 (33.4)	152 (24.7)	135 (27.2)	1e-04
Negative	1027 (66.6)	463 (75.3)	361 (72.8)	

LVI: Lymphovascular Invasion. SN: Sentinel Node. No:Number. IHC: Immuno-Histochemistry. FISH: Fluorescent In Situ Hybridization.

**Table 2:** Clinical and pathological features of the sentinel node negative and positive tumours.

Training Set	Negative SNs *Number(%)*	Positive SNs *Number(%)*	Chi^2^ pvalue
**All patients**	1027 (100)	516 (100)	
**Age (years)**			
<60	608 (62.5)	365 (37.5)	
60–65	150 (68.8)	68 (31.2)	p<0.0001
>65	269 (76.4)	83 (23.6)	
**ER (IHC)**			
Positive	879 (65.5)	464 (34.5)	p = 0.0168
Negative	148 (74)	52 (26)	
***HER2*** ** (IHC-FISH)**			
Positive	85 (64.4)	47 (35.6)	p = 0.58
Negative	942 (64.7)	469 (33.3)	
**ER ** ***HER2*** **(IHC-FISH)**			
ERneg HER2neg	117 (79.6)	30 (20.4)	
ERpos HER2neg	825 (65.3)	439 (34.7)	p = 0.0031
ERpos HER2pos	54 (68.4)	25 (31.6)	
ERneg HER2pos	31 (58.5)	22 (41.5)	
**Tumour size (mm)**			
≤20	938 (70.4)	395 (29.6)	p<0.0001
>20	70 (37.4)	117 (62.6)	
**Histological Grade**			
1	363 (71.6)	144 (28.4)	
2	447 (63.2)	260 (36.8)	p = 0.0083
3	207 (65.1)	111 (34.9)	
**Mitotic index**			
1	719 (68.6)	329 (31.4)	
2	140 (59.1)	97 (40.9)	p = 0.013
3	150 (63.8)	85 (36.2)	
**LVI**			
Negative	851 (73.1)	313 (26.9)	p<0.0001
Positive	160 (44.4)	200 (55.6)	
**Subtype**			
Ductal	847 (65.5)	446 (34.5)	
Lobular	143 (70.4)	60 (29.6)	p = 0.076
Other(s)	37 (78.7)	10 (21.3)	
**No SN removed**			
1	287 (27.3)	129 (31)	
2–3	470 (45.7)	320 (40.5)	p<0.0001
>3	270 (26)	67 (19.9)	

LVI: Lymphovascular Invasion. SN: Sentinel Node. No:Number. IHC: Immuno-Histochemistry. FISH: Fluorescent In Situ Hybridization.

**Table 3:** Multivariate logistic regression model to predict the probability of axillary metastases.

		Training Set. Institut Curie				Validation Set. Institut Curie				Validation Set. Tenon Hospital			
		N	OR	CI	pvalue	N	OR	CI	pvalue	N	OR	CI	pvalue
**Tumor Size**	**mm**	1543	1.08	1.O6/1.1	<0.00001	615	1.07	1.04/1.1	<0.00001	496	1.04	1.01/1.06	0.005
**LVI**	**No**	1164	1			502	1			386	1		
	**Yes**	360	2.69	2/3.5	<0.00001	113	4.14	2.6/6.6	<0.00001	110	5.2	3.2/8.5	<0.00001
**Molecular Subtypes**	**ERneg HER2neg**	147	1			40	1			24	1		
	**ERneg HER2pos**	53	2.95	1.4/6.3	0.005	18	3.8	0.9/16	0.07	16	12.5	1.6/95	0.01
	**ERpos HER2neg**	1264	2.9	1.8/4.6	0.00001	528	2.4	0.9/6.6	0.08	347	9.3	1.6/51.9	0.01
	**ERpos HER2pos**	79	1.8	0.9/3.5	0.08	29	2	0.55/7.8	0.27	109	8.2	1.4/47.4	0.02
**Age**	**< = 60 (years)**	973	1			330	1			296	1		
	**]60–65]**	218	0.78	0.5/1.1	0.16	90	0.77	0.4/1.4	0.4	60	1.3	0.7/2.5	0.32
	**>65**	352	0.56	0.4/0.7	0.0001	195	0.6	0.4/1.03	0.06	140	0.6	0.3/0.9	0.04

LVI: Lymphovascular Invasion. ERneg: Estrogen Receptor Negative. ERpos: Estrogen Receptor Positive. HER2neg: HER2 negative. Her2pos: HER2 positive. OR: Odds Ratio. CI: Confidence Interval. N: Number of patients

**Table 4:** Number and percentage of lymph node negative breast cancer patients by molecular subgroup as determined by a combination of ER and *HER2* IHC markers. Review of literature.

References	Molecular Subtypes				ER status
	ER- HER2-LNN/LN (%)	ER+ HER2-LNN/LN (%)	ER- HER2+LNN/LN (%)	ER+ HER2+LNN/LN (%)	
**Liu et al** [11]	237/477 (49.6)		68/149 (45.6)		HR negative samples
**Lu et al** [9]	27/38 (71)	25/60 (41.6)	6/15 (40)	6/16 (37.5)	Threshold 10%
**Kim et al** [14]	118/237 (49.7)	171/345 (49.6)	61/133 (45.9)	23/61 (37.7)	Threshold 10%
**Nguyen et al** [15]	59/89 (66)	440/595 (74)	16/32 (50)	49/77 (64)	Threshold 1%
**Crabb et al** [10]	199/315 (63.3)	1397/2397 (58.5)	109/240 (32)	95/210 (45.2)	Threshold 1%
**Van Calster et al** [12]	126/193 (65.3)	1139/1778 (64)	56/88 (63.6)	83/168 (49.4)	Any positive nuclear staining
**Lee et al** [13]	33/58 (56.8)	143/189 (75.6)	27/41 (65.8)	38/54 (70.4)	Threshold 10%
**Voduc et al** [16]	350/556 (62.9)	1154/2017 (57.2)	102/227 (44.9)	85/185 (45.9)	ER positive status. Threshold 1%

LNN; Nulber of Lymph Node Negative Patients. LN; Number of patients.

**Table 5:** Percentage and Confidence Interval of positive sentinel node calculated for each 5 mm tumour size subclasses from 0 to 40 mm.

Molecular Subtypes	ERneg HER2neg		ERneg HER2pos		ERpos HER2pos		ERpos HER2neg	
Tumour Size (mm)	SNpos/SN	% CI	SNpos/SN	% CI	SNpos/SN	% CI	SNpos/SN	% CI
]0,5]	0/13	0 (0–24)	3/8	35.5 (8–75)	1/12	8.3 (0.2–38)	7/106	6.6 (3–13)
]5,10]	4/35	11.4 (3–26)	4/20	20 (5–43)	9/37	24.3 (12–41)	138/677	20.4 (17–23)
]10,15]	9/59	15.2 (7–27)	9/26	34.6 (17–55)	24/91	26.3 (17–36)	221/726	30.4 (27–33)
]15,20]	11/60	18.3 (9–30)	9/18	50 (26–74)	16/46	34.7 (21–50)	155/375	41.3 (36–46)
]20,25]	7/27	25.9 (11–46)	7/8	87.5 (47–99)	7/13	53.8 (25–80)	73/122	59.8 (50–68)
]25,30]	1/5	20 (0.5–71)	2/2	100 (16–100)	7/9	77.7 (40–97)	22/38	57.9 (40–73)
]30,35]	1/1	100 (2.5–100)	NA	NA	2/3	66.6 (9–99)	11/19	57.9 (33–79)
]35,40]	2/2	100 (15–100)	1/1	100 (25–100)	2/2	100 (15–100)	12/16	75 (29–96)

ERneg; ER negative status. ERpos; ER positive status. HER2neg; HER2 negative status. HER2pos; HER2 positive status. SNpos; number of positive sentinel node. SN; number of sentinel node retrieved.

## Discussion

The aim of our work was to decipher the relation between the molecular subtype classification as defined by a combination of *ER* and *HER2* status and the probability of a positive sentinel node biopsy. Using one training set and two validation sets we showed a benefit to introduce the *ER* and *HER2* biomarkers interaction covariate to identify, before surgery, a patient with a high risk of axillary metastasis. Using tumour size, lympho-vascular invasion, molecular subtypes classification and age at diagnosis, we designed a robust multivariate logistic regression model to determine the probability of having a positive sentinel node biopsy. We validated this model in two independent and very different datasets and showed a very similar performance in terms of calibration and discrimination. Lu et al identified a similar multivariate model to predict lymph node metastases that included tumour size, lympho vascular invasion and tumour subtypes defined by a combination of ER status, *HER2* status and modified Bloom and Richardson grade [9]. Furthermore we identified for each molecular subtype a very specific correlation pattern between the tumour size and the probability of a positive sentinel node biopsy. The ER negative *HER2* negative breast cancer subgroup nodal status was almost independent from the tumour size with a relative constant trend of axillary metastases around 20%. Conversely the ER positive or *HER2* positive breast cancer subgroups showed a strong and almost linear correlation between the tumour size and the percentage of axillary metastasis.

Tumour size and lympho vascular invasion are the main predictors of axillary metastases identified in many studies [17]–[25]. However tumour size and lympho vascular invasion have never been robustly related to any pathological or biological marker. High throughput gene expression profiles analysis failed to identify a set of genes correlated to the nodal status, the tumour size or the lympho vascular invasion [9]. The gene expression profile of paired primary tumour and corresponding axillary metastases have previously been shown as very similar [30]. From these observations, conclusions have been drawn that growth rate, time and stochastic factors seem to be the main determinants of the nodal status. However, several authors have recently underscored a significant relation between the molecular subtypes classification and the axillary status of breast cancer patients [9]–[16]. These evidences sustained the idea that nodal status is still a potential signature of the intrinsic biological properties of a primary tumour. Perou et al have identified the molecular subtype classification in the late 90′ and it was a major breakthrough in the breast cancer research process [1]. This classification underscored the great heterogeneity of breast cancer. It is now a common knowledge that the pathologic characteristics, the aCGH profiles, the gene and miRNA expression profiles and altered pathways are dramatically different between these categories and sustained an overview of breast cancer as a disease composed of very different and independent molecular subgroups.

For each molecular subtype as defined by a combination of ER and *HER2* immuno-histochemistry markers, we summarized (table 5) eight publications addressing the percentage of axillary metastases [9]–[16]. As reported in our study the ER negative *HER2* negative tumour subgroup has the lowest rate of axillary metastasis and the *HER2* positive tumour one, the highest. We hypothesized from the whole results that the axillary lymph node metastasis process is predominantly related to intrinsic biological properties in the ER negative *HER2* negative breast cancer subgroup when stochastic events, tumour size, growth rate and lympho vascular invasion are the main determinants in both the ER positive or the *HER2* positive breast cancer subgroups. As the molecular subtypes differ in terms of relapse free survival and overall survival [ER negative *HER2* negative and *HER2* positive breast cancer patients experience a shorter relapse free survival and overall survival] and the nodal status is the strongest prognostic predictor, we highlighted a very complex interaction network between the primary tumour, the nodal status and the distant metastases. The molecular subtype classification is one determinant of this network.

Finally we showed that biologically-driven analyses are able to build new models with higher performance in terms of breast cancer axillary status prediction. The molecular subtype classification is the first stratification level of breast carcinoma and strongly interacts with the axillary and distant metastasis process. Large integrative analyses have to be performed to explain why ER negative *HER* negative tumours have a low rate of axillary metastasis and a high rate of distant metastases. Conversely *HER2* positive tumours have a rate of axillary metastases strongly related to the tumour size and a high rate of distant metastases.
